# Preventing spread of the invasive spotted lanternfly via texture-based automated egg detection

**DOI:** 10.3389/finsc.2026.1678964

**Published:** 2026-03-23

**Authors:** Karla Negrete, Rhys Butler, Emily Wallis, Emily Magnani, Melissa Benzinger Mcglynn, Matthew McDonald, Nicolas J. Alvarez, Maureen Tang

**Affiliations:** 1 Department of Mechanical Engineering & Mechanics, Drexel University, Philadelphia, PA, United States; 2 Department of Chemical & Biological Engineering, Drexel University, Philadelphia, PA, United States; 3 United States Department of Agriculture (USDA) Forest Pest Methods Laboratory, Marketing and Regulatory Programs, Animal and Plant Health Inspection Service (MRP-APHIS), Buzzards Bay, MA, United States; 4 Port of Wilmington, MRP-APHIS, Wilmington, DE, United States

**Keywords:** computer vision, egg mass detection, entomology, invasive species, spotted lanternfly, support vector machine, surveillance, texture features

## Abstract

The invasive spotted lanternfly (*Lycorma delicatula*) threatens U.S. agriculture, particularly grape and tree fruit production. Early detection of egg masses is critical for limiting spread, yet current surveillance relies heavily on manual inspection, which is labor-intensive and difficult to scale. The lanternfly spreads primarily through human-assisted transport pathways, including trains, trucks, and freight infrastructure, enabling long-distance dispersal of egg masses. Here, we present a proof-of-concept automated image classification pipeline for SLF egg mass detection based exclusively on spatial texture features. Using a curated laboratory image dataset and descriptors including Gray-Level Co-occurrence Matrix (GLCM), GLDS (Gray Level Difference Statistics), and Hu and Zernike moments, we implemented a feature filtering and selection strategy to construct an interpretable, low-dimensional model. The final image-level screening classifier, a support vector machine with a radial basis function kernel trained on 12 selected features, achieved a mean Matthews Correlation Coefficient (MCC) of 0.881 (SD 0.037) under 5-fold stratified cross-validation. Generalization performance was evaluated on a held-out test set using bootstrap resampling (1,000 iterations), yielding a mean MCC of 0.836 (SD 0.037; 95% CI: 0.761–0.904). This image-level proof-of-concept under controlled imaging demonstrates that low-cost, scalable, and interpretable texture-based computer vision approaches may provide reliable early detection of SLF egg masses, supporting human-in-the-loop surveillance efforts in high-risk transport corridors and improving cost and reliability over manual inspection workflows.

## 1 Introduction

The spotted lanternfly (SLF, *Lycorma delicatula*) is a high-priority invasive pest in the United States. Native to Asia, SLF causes damage by feeding on phloem and indirectly by producing honeydew that promotes mold growth (1). First detected in Pennsylvania in 2014, SLF has rapidly spread to at least 14 states, reaching as far west as Illinois University (2). The economic impact is substantial, with Pennsylvania alone facing $7.9 million in grape revenue losses (1). Broader agricultural sectors are also at risk, with $915 million in the grape and tree fruit industries vulnerable where SLF is established (3). The pest’s expansion is concentrated along major transport routes, especially highways and rail lines, where its preferred host, Tree of Heaven (*Ailanthus altissima*), is prevalent.

The egg stage of SLF plays a key role in its long-distance dispersal. Egg masses, laid in early fall and containing 27–35 eggs, are camouflaged beneath a brown-gray waxy ootheca (4, 5). As the longest and most dormant life stage, eggs overwinter and can hatch as late as June. Their habit of ovipositing on a wide range of natural and artificial substrates, combined with their low visual detectability, increases the risk of accidental transport via freight and vehicles (1). Given their potential for long-distance spread, primarily through human-mediated transport, SLF threatens key agricultural regions, including California’s grape-producing areas, projected to be impacted by 2033 (6). This rapid spread highlights the need for surveillance at critical entry points such as ports, rail networks, and state borders to intercept further introductions across the US (7). For example, California has established border protection stations along major highways to regulate the movement of invasive species (8). Meanwhile, SLF-quarantined states in the East, like New Jersey and Pennsylvania, mandate inspection protocols focused on rail networks (9, 10). Current detection efforts are heavily based on manual inspections of vehicles, cargo, and rail cars to locate and remove egg masses. Complementary methods such as trapping (11–13), environmental DNA (eDNA) analysis (14), and scent-detection dogs (4) are in use but remain labor-intensive and unsuitable for large-scale deployment.

Automated image analysis offers a promising solution, with advances in machine learning and computer vision enabling new approaches for invasive species detection (15). These technologies have been successfully applied across diverse taxa, including plants (16, 17), zooplankton (18), and corals (19), through classification of visual features such as shape and texture. While commercial applications remain limited, several research tools and open-source platforms exist. Building on these successes, computer vision provides a scalable framework for automating the visually distinct task of SLF egg mass inspection at transportation checkpoints. Many computer vision methods face challenges due to limited data. Deep learning requires large, diverse datasets and extensive computational resources, often struggling with generalization and deployment constraints (20, 21).

In contrast, texture analysis offers an interpretable approach that reduces data complexity during model training. The complex surface morphology of SLF egg masses generates rich textural signals essential for precise detection (22, 23). Texture image retrieval uses spatial domain feature extraction, capturing gray-level intensity relationships among pixels (24). Texture is commonly modeled as primitive elements (“texels”) arranged spatially to define surface appearance (25). Statistical descriptors quantify these local patterns, allowing the differentiation of complex surfaces and forming the basis for an effective automated classification (26). Given the success of combining textural features with classification across various applications (27–31), this study explores their potential to streamline SLF egg mass detection and ease the burden of manual inspections.

To demonstrate proof of concept, we trained a simple support vector machine (SVM) classifier on 100 extracted texture features to distinguish images with SLF egg masses from those without (**Table 1**). Our dataset comprises experimentally collected images of egg masses on multiple substrates, captured from two angles to reflect real-world variation. This was augmented with curated public texture datasets that represent materials common to freight and rail infrastructure. Our study has two aims: first, to establish a straightforward, texture-based approach to egg mass detection that accounts for practical imaging constraints; and second, to provide a publicly accessible dataset of over 300 annotated egg mass images with computed texture features to support ongoing development of computer vision methods for SLF detection. Given the urgent need for scalable detection tools, this work pioneers the application of 79 texture-based classification for identifying SLF egg masses.

**Table 1:** Overall SVM classification performance on the curated SLF dataset.

Dataset	MCC	F1 (%)	Prec. (%)	Rec. (%)	ROC/PR
Training (CV)	0.881 ± 0.037	98.7	98.6	98.7	0.999/0.984
Validation (CV)	0.838 ± 0.037	97.0	96.6	96.7	0.989/0.937
Held-out test	0.836 ± 0.037	97.0	96.5	96.6	0.987/0.934
External citizen science	0.060 (–0.016–0.134)	5.9 (2.2–10.1)	5.8 (2.2–10.1)	6.1 (2.3–10.2)	0.71/0.059

Metrics are reported as mean ± SD over 5-fold stratified cross-validation. For the held-out test set, values represent bootstrap means with 95% confidence intervals (1,000 resamples). Abbreviations: MCC, Matthews correlation coefficient; F1, F1-score; Prec., precision; Rec., recall; ROC, receiver operating characteristic AUC; PR, precision–recall AUC.

## 2 Experimental methods and procedures

### 2.1 Dataset composition

**Figure 1:**
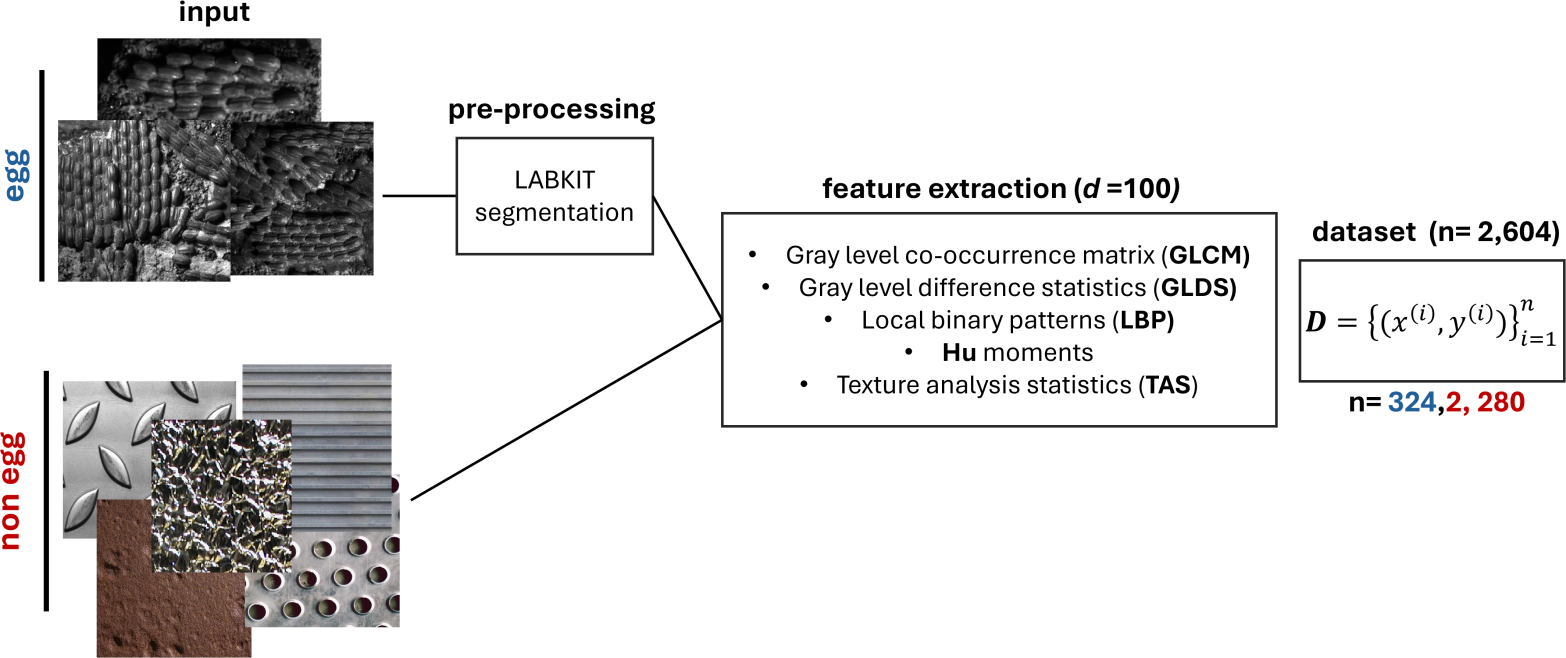
Dataset creation pipeline illustrating assembly of *egg* and *non-egg* image classes from multiple sources, including public texture databases.

The dataset comprises 3,129 grayscale images, with 327 labeled as the *egg* class and 2,802 labeled as *non-egg*, resulting in a class imbalance ratio of approximately 1:7. The dataset creation pipeline is illustrated in **Figure 1**. Non-egg images are derived from publicly available texture databases that simulate the surfaces of freight railcars and containers. Ten texture classes were selected from the Describable Textures Dataset (DTD) (32): cracked, flecked, grid, grooved, honeycombed, meshed, perforated, pitted, porous, and wrinkled. The dataset is complemented by the Severstal Steel Defect Detection dataset, which comprises high-resolution grayscale images of steel surfaces annotated for four defect types (33).

Egg images originate from a USDA trapping study in Odessa, Delaware (34), comprising 1,072 egg masses collected using lampshade traps deployed by APHIS PPQ. Each egg mass typically contains 30–50 aligned eggs, and many images contained multiple egg masses. For the purposes of this study, model splitting units are images, not individual eggs or egg masses. Because all eggs were collected from the same site at the same time, source or session grouping was not available. Images were captured using an Alvium G1–131 monochrome camera with a 12-mm Moritex lens at a working distance of 8 inches, illuminated by directional white LEDs (SL2420, Advanced Illumination). The location and angle of the light source were maintained consistently across all samples, and other light sources were eliminated from the room in which samples were imaged. Resolution was 1450 × 694 pixels with a 50-ms exposure. Two imaging angles were used: 0° (direct) and 45° (angled). Metadata includes imaging angle, egg count, and surface type.

An independent dataset was used for external validation to assess model performance under unconstrained, real-world imaging conditions. The positive class of this dataset consisted of 251 citizen science images of SLF egg masses obtained from web scraping and a previous outreach effort. Porter (35) Images exhibited substantial variability in season, substrate, illumination, working distance, focal length, and acquisition device. The presence of SLF egg masses in each image was verified manually, as citizen science submissions frequently contained incomplete or erroneous metadata. Several examples are shown in SI.

### 2.2 Segmentation and pre-processing

Egg masses were segmented using Labkit (36), a supervised pixel-classification tool integrated within FIJI. Random forest classifiers were trained on manually annotated images to distinguish egg mass foregrounds from the background. Two angle-specific classifiers (0° and 45°) were applied batch-wise through a custom FIJI macro, incorporating histogram equalization and standardized output formatting. An example of this workflow is shown in **Supplementary Figure 1**, applied to a direct field image. The top panel displays the original image, the center panel presents the Labkit segmentation, and the bottom panel illustrates the delineation of egg masses from the complex background, which consists of a lampshade layered over a metal substrate. Morphological operations such as hole filling and small-object removal refine the binary masks. Bitwise masking extracts *egg* mass regions for subsequent feature extraction. Because feature extraction consists exclusively of textural analyses of pixel intensity patterns, morphology effects due to segmentation should not impact the model performance. However, to reduce the possibility of pipeline artifacts, each negative image was also segmented using a randomly assigned segmentation pattern from the 327 available egg masks. This control does not ensure content-symmetric windowing, but approximates symmetry to reduce the possibility of morphological image artifacts and ensure that the learning pipeline distinguishes based on genuine texture differences.

### 2.3 Feature extraction

All images are processed using PyFeats (37) to extract 100 grayscale texture features per image across five categories: Gray-Level Co-occurrence Matrix (GLCM), Local Binary Patterns (LBP), Gray-Level Difference Statistics (GLDS), Hu and Zernike moments, and Threshold Adjacency Statistics (TAS). A concise description of these features is provided in **Table 2**. Each image is represented as a feature vector x*_i_* ∈ R^100^, paired with a binary label *y_i_* ∈ {0,1} indicating *egg* (1) or *non-egg* (0).

**Table 2:** Summary of texture-descriptor families extracted using PyFeats (37).

Category	Description	Representative features
GLCM (38)	Second-order statistics describing spatial gray-level dependencies within local neighborhoods	Contrast, Correlation, Angular Second Moment, Inverse Difference Moment, Entropy, Variance
GLDS (39)	Absolute gray-level difference statistics computed across multiple directions	Homogeneity, Energy, Entropy, Mean
LBP (40)	Local binary encodings capturing fine-scale texture and local contrast	Entropy, Energy
Hu Moments (41)	Invariant moment descriptors capturing global shape properties independent of rotation and scale	Hu moment 1
Zernike Moments (41)	Orthogonal moment descriptor computed over the unit disk, capturing rotationally invariant global structure	Low-order Zernike moments

These descriptor classes quantify spatial, structural, or intensity-based patterns in the images. GLCM, Gray-level co-occurrence matrix; GLDS, gray-level difference statistics; LBP, local binary patterns; Hu, invariant image moments; TAS, texture adjacency spectrum features are listed with their corresponding definitions.

### 2.4 Data preparation

The dataset is split into training (80%) and testing (20%) subsets using stratified sampling to preserve the *egg*/*non-egg* proportions. Feature selection employs a hybrid approach. First, Pearson correlation filtering is applied exclusively to the training set to remove collinear features with an absolute pairwise correlation exceeding |*ρ*| *>* 0.70. These features are then dropped from the validation set for consistency. Second, supervised univariate feature selection retains features with the strongest statistical association with the target class, as determined by ANOVA F-statistics, which reflects discriminatory power based on between-class versus within-class variance.

### 2.5 Model training

The SVM classifier utilizes class weights that are inversely proportional to class frequencies to address class imbalance. The minority *egg* class receives higher weight, computed as:


$$w_{c}=\frac{n_{samples}}{n_{classes}\cdot n_{c}}$$

The pipeline involves standardizing features to have a mean of zero and a variance of one, performing SelectKBest dimensionality reduction, and classifying using an SVM. SVMs are chosen for their strong performance on small to moderate datasets and ability to model complex, nonlinear boundaries with limited data (42). Their robustness and capacity to generalize well, especially when combined with regularization and kernel functions, make them suitable for this task, given the modest sample size and class imbalance (43–45). where *c* indexes the class label, *n_c_* is the sample count in class *c*, *n*_samples_ = 3,129, and *n*_classes_ = 2. This imbalance makes errors on *egg* images roughly 10 times more costly, encouraging sensitivity to the minority class.

#### 2.5.1 Support vector machines

SVMs identify a separating hyperplane that maximizes the margin between classes (46). For linearly separable data, this solves:


$$\underset{w,b}{\min}\;\frac{1}{2}\|w{\|}^{2}\text{ s}.\text{t}.\;y_{i}(w\cdot x_{i}+b)\geq 1,\;i=1,\ldots ,n.$$

To handle nonlinearly separable data, slack variables *ξ_i_* ≥ 0 allow margin violations, yielding the soft-margin problem:


$$\underset{w,b,\xi }{\min}\;\frac{1}{2}\|w{\|}^{2}+C\sum_{i=1}^{n}\xi_{i}\text{ s}.\text{t}.\;y_{i}(w\cdot x_{i}+b)\geq 1-\xi_{i},\;\xi_{i}\geq 0.$$

Kernels implicitly project inputs to higher-dimensional spaces, enabling linear separation (47). Common kernels include linear $\;K(x_{i},x_{j})=x_{i}^{⊤}x_{j}$; polynomial $K(x_{i},x_{j})={\left(\gamma x_{i}^{⊤}x_{j}+r\right)}^{d}$; and radial basis function (RBF) $K(x_{i},x_{j})=\exp(-\gamma \|x_{i}-x_{j}{\|}^{2})$. Kernels impose inductive biases capturing structures of varying complexity. Key hyperparameters include regularization C, kernel sensitivity γ, polynomial degree d, and feature selection parameter k, the latter defining the number of features selected by ANOVA F-statistics before fitting.

Hyperparameters were optimized via 5-fold stratified cross-validation to ensure robust generalization (48). Performance was evaluated using the Matthews Correlation Coefficient (MCC). Feature selection was performed using SelectKBest, with the number of features k∈{4,8,12,15}. The support vector classifier (SVC) search space included kernels {linear,rbf,poly}, regularization parameters C∈{0.1,1,2,3,4,5}, and γ∈{scale,0.1} for the RBF kernel. For the polynomial kernel, degrees d∈{2,3} were considered with coef0=0. The best model selected by cross-validation was retrained on the full training set and subsequently evaluated on an independent test set. Experiments use scikit-learn 0.24 (49).

#### 2.5.2 Model evaluation

Metrics are derived from the elements of the confusion matrix: true positives (TP), false positives (FP), true negatives (TN), and false negatives (FN). Accuracy, while intuitive, can be misleading in imbalanced data dominated by *non-egg* samples. More informative metrics include:


$$\text{Recall}=\frac{TP}{TP+FN},\text{ Precision}=\frac{TP}{TP+FP}$$

Recall measures the fraction of actual *egg* textures correctly identified, and precision measures the correctness of predicted *egg* textures. The F-score balances recall and precision:


$$F_{1}=\frac{2\cdot \text{Precision}\cdot \text{Recall}}{\text{Precision}+\text{Recall}}=\frac{2TP}{2TP+FP+FN}$$

MCC provides a balanced measure robust to class imbalance Chicco and Jurman (50, 51):


$$\text{MCC}=\frac{TP\cdot TN-FP\cdot FN}{\sqrt{\left(TP+FP)(TP+FN)(TN+FP)(TN+FN\right)}}$$

MCC ranges from −1 (inverse prediction) to 1 (perfect prediction), with 0 indicating random performance.

Threshold-independent metrics include the receiver operating characteristic (ROC) curve and the precision-recall curve (PRC). The ROC curve plots the actual positive rate against the false positive rate across thresholds, with the area under the curve (AUC) summarizing performance (52, 53). However, ROC curves may mask poor performance in imbalanced data. PRC curves, plotting precision versus recall, provide a more sensitive evaluation under class imbalance (54). Together, these metrics provide a comprehensive assessment of the situation. Model performance is estimated using 5-fold stratified cross-validation, which yields a robust and unbiased evaluation while mitigating overfitting. To determine the goodness of fit of the final SVM model, bootstrapping is applied with 1000 bootstrap replicates in order to establish a suitable gaussian fit and accurately estimate confidence intervals.

## 3 Results

### 3.1 Dimensionality reduction and feature selection

Feature selection is critical in SVM modeling to improve generalization, reduce complexity, and enhance interpretability (55), particularly for high-dimensional, imbalanced datasets where classifiers often favor the majority class at the expense of minority-class performance (48, 56–59). Targeted feature selection combined with imbalance-aware learning mitigates these effects.

A two-step filter-based approach was applied to reduce redundancy and improve model stability. Pearson correlation filtering removed collinear features using |*ρ*| *>* 0.70 (approximately 49% shared variance, *R*^2^ = 0.49) (60–62), reducing 100 texture features to 43 (**Supplementary Figure 3**). Univariate ANOVA F-statistic ranking (SelectKBest) then retained the top 12 features, balancing overfitting from high dimensionality against underfitting from excessive pruning (**Supplementary Figure 4b**).

Mean MCC across 5-fold cross-validation peaked at 0.881 (SD 0.037) for 12 features, supporting this selection as optimal. The final feature set (**Supplementary Figure 6**) spans GLCM, Hu moments, GLDS, and Zernike moments. The top-ranked features are dominated by GLCM and GLDS descriptors, capturing complementary information on gray-level spatial arrangements and intensity differences. Together with lower-ranked Hu and Zernike moments, these features encode fine-grained texture, spatial, and shape characteristics that enable discrimination of granular SLF egg mass textures from more uniform non-egg backgrounds.

### 3.2 Model selection and optimization

SVM models were trained on feature subsets following correlation filtering to assess dimensionality effects. Performance, measured by MCC and averaged over 5-fold cross-validation, increased with feature count, peaked at 12 features (MCC 0.881, SD 0.037), and declined thereafter (**Supplementary Figure 4b**).

Hyperparameters were optimized using a grid search over 120 configurations spanning linear, RBF, and polynomial kernels, evaluated with 5-fold cross-validation (600 total fits). MCC guided model selection (51). The RBF kernel consistently outperformed alternatives (**Supplementary Figure 5a**), reflecting its capacity to model subtle nonlinear decision boundaries. The optimal configuration (*C* = 5, *γ* = scale) was identified using the 12 selected features. The final pipeline integrates standard scaling, SelectKBest feature selection, and the tuned SVM classifier.

### 3.3 Performance evaluation and visualization

The final SVM model (RBF kernel, *C* = 5, *γ* = scale) trained on the 12 selected features achieved a mean MCC of 0.881 (SD 0.037) under 5-fold stratified cross-validation, with fold-wise MCC values ranging from 0.822 to 0.939. Mean fit and score times were 0.265 ± 0.057 s and 0.021 ± 0.004 s per fold, respectively, indicating efficient training and inference.

Generalization performance was evaluated on a held-out test set using bootstrap resampling (1,000 iterations), yielding a mean MCC of 0.836 (SD 0.037; 95% CI: 0.761–0.904).

**Figure 2:**
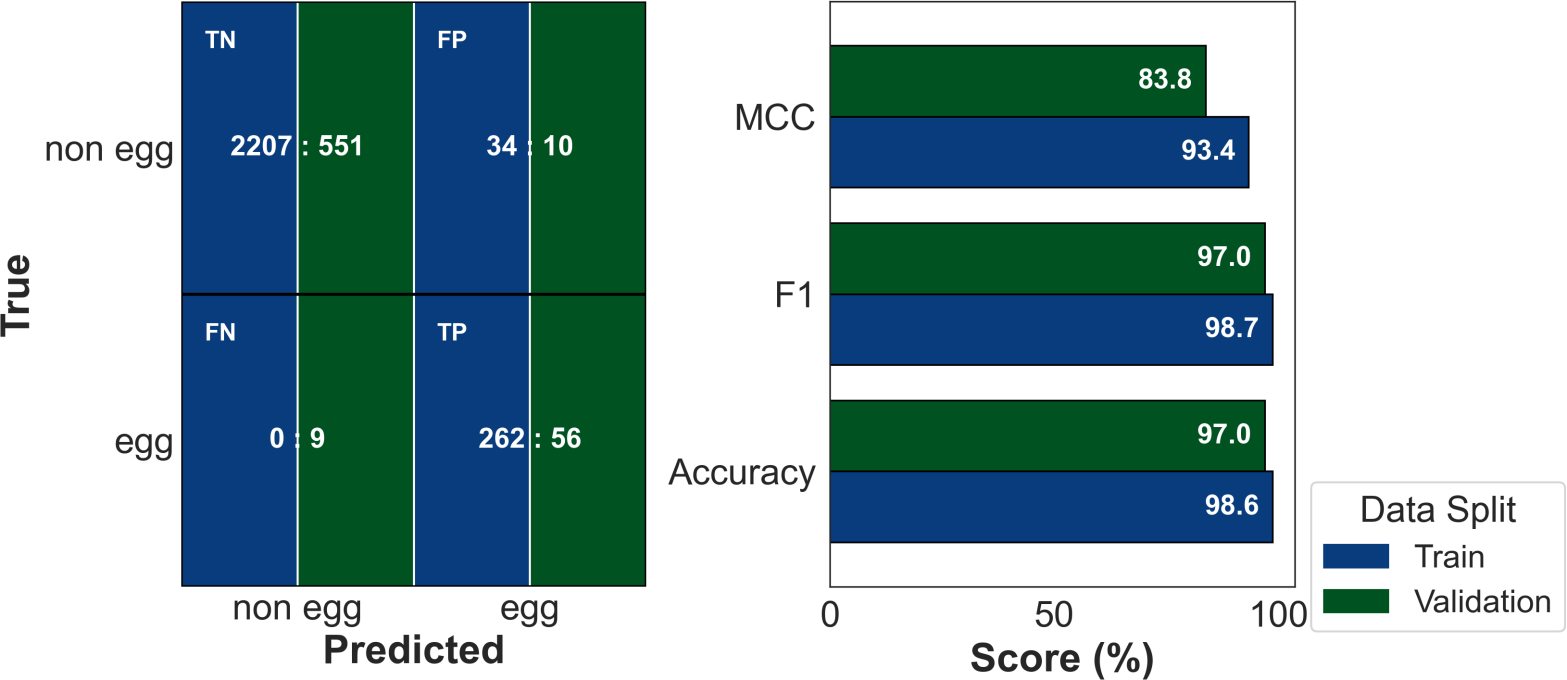
Final SVM classification performance, including confusion matrix and classwise precision.

The confusion matrix (**Figure 2**) demonstrates stable classification under class imbalance. On the training set, the model produced 2,207 true negatives, 34 false positives, zero false negatives, and 262 true positives. Validation results were similarly strong, with 551 true negatives, ten false positives, nine false negatives, and 56 true positives. Accuracy, F1-score, and MCC reached 98.6%, 98.7%, and 93.4% on training, and 97%, 97%, and 83.8% on validation, respectively (**Table 3**).

**Table 3:** Class-wise performance metrics and confusion matrix counts for the training and validation sets.

Dataset	Class	Precision	Recall	F1	PR AUC	TP	FP	FN	TN
Training	Egg Non-egg	0.885 1.000	1.000 0.985	0.939 0.992	0.984 –	262 2207	34 0	0 34	2207 262
Validation	Egg Non-egg	0.848 0.984	0.862 0.982	0.855 0.983	0.937 –	56 551	10 9	9 10	551 56

Precision–recall AUC (PR AUC) is reported for the egg (minority) class.

**Figure 3:**
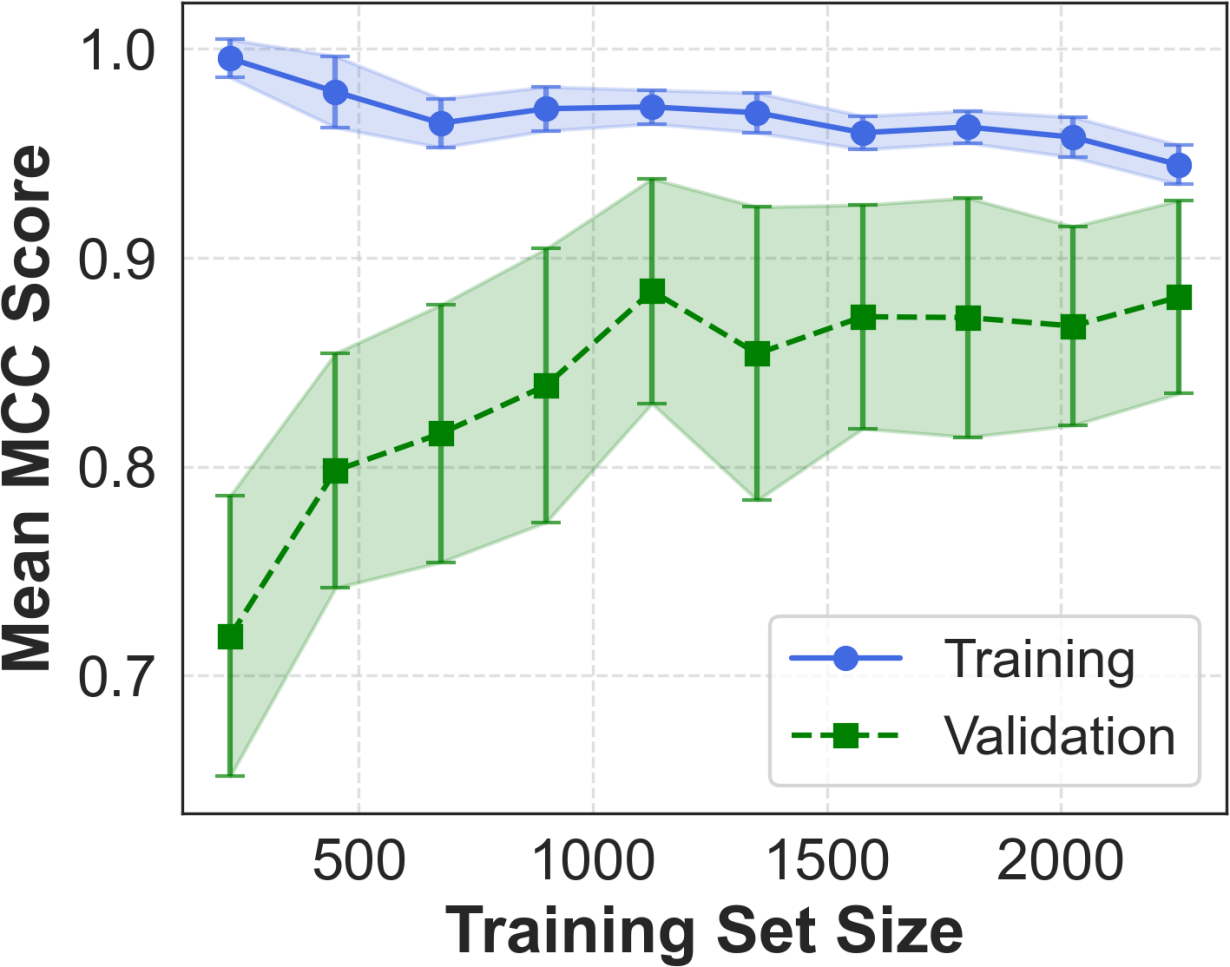
Learning curves for the optimized SVM. Mean MCC increases with training set size and converges between training (blue) and cross-validation (green) performance. Shaded regions denote fold to-fold variability, indicating stable generalization and minimal overfitting. Error bars indicate 95 percent confidence intervals.

**Figure 4:**
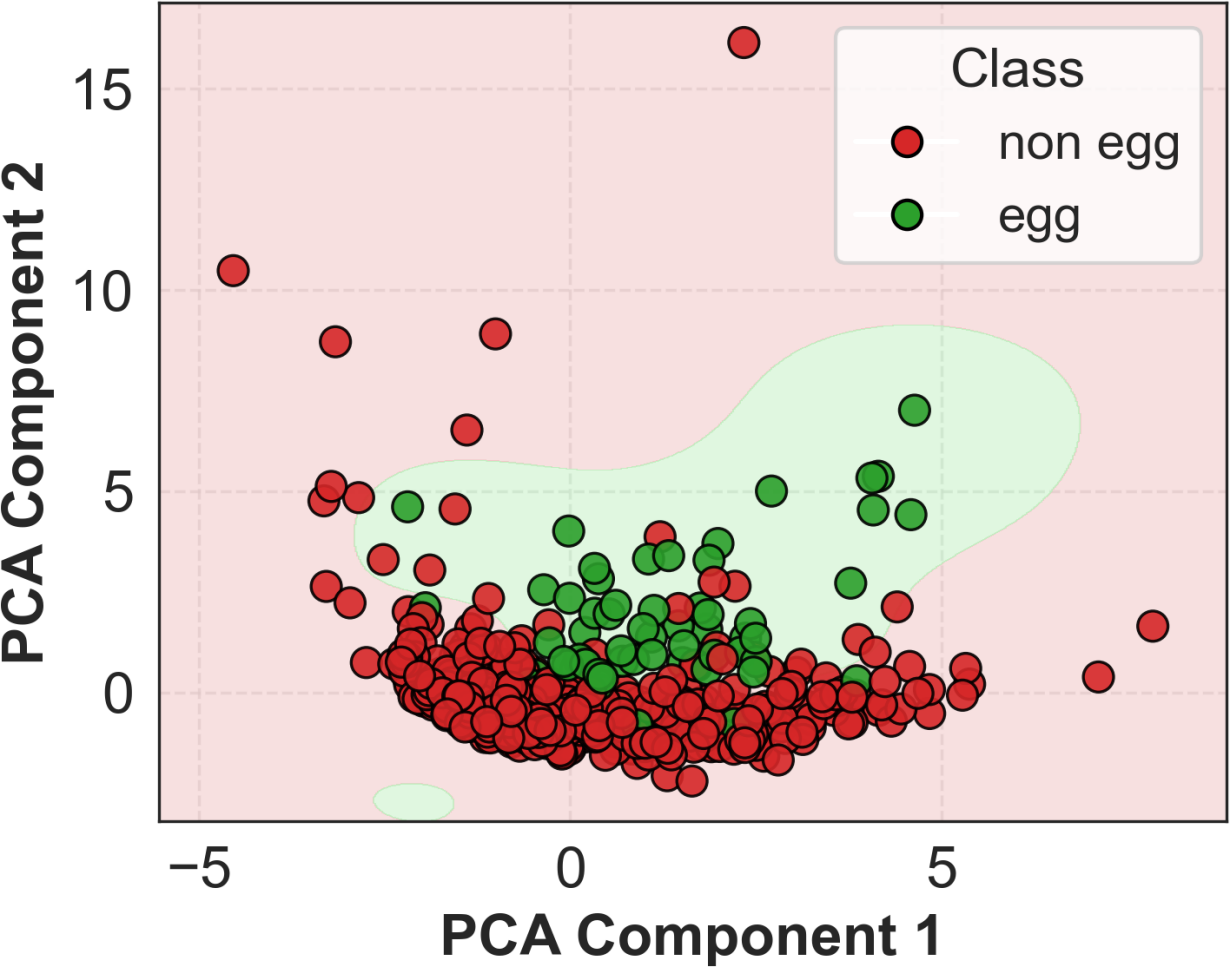
PCA of the top 12 features. The 2-D projection shows partial class clustering, supporting feature discriminability.

Learning curves (**Figure 3**) show rapid convergence and minimal overfitting. ROC and precision–recall curves further confirm strong discriminative performance (**Supplementary Figure 7**), with training AUCs of 0.999 (ROC) and 0.984 (PR), and validation AUCs of 0.989 (ROC) and 0.937 (PR). To preserve interpretability, PCA was not used during training but applied *post hoc* to the 12-feature space; the resulting 2D projection (**Figure 4**) reveals partial class separation, supporting the discriminative power of the selected features.

When applied to the external citizen science dataset, the classifier exhibited substantially reduced performance, with an accuracy of 0.70 (95% CI: 0.668–0.730), F1-score of 0.059 (95% CI: 0.022–0.101), and MCC of 0.060 (95% CI: –0.016–0.134). This near-chance MCC indicates lack of external validity under heterogeneous conditions. Differences between the external images and the curated laboratory dataset include variation in angle working distance, illumination, and substrate composition, all of which shift the resulting feature distributions substantially outside the training domain.

## 4 Discussion

Automated detection of SLF egg masses along transit networks is uniquely challenging due to their visually subtle, highly textured appearance. This study shows that a conventional image classification approach can reliably distinguish egg masses from diverse background substrates under controlled laboratory conditions when guided by targeted texture descriptors. Given the small, imbalanced dataset, careful validation was essential to ensure that performance reflected meaningful texture-based separation rather than overfitting. Initial models trained on all 100 descriptors showed poor generalization, with perfect training accuracy and much lower validation scores. Non-converging learning curves revealed persistent train–test gaps, consistent with the curse of dimensionality, where an excessive feature-to-sample ratio leads to memorization rather than generalization (63). Redundant or noisy features further increased model complexity and variance, underscoring the value of feature selection to improve robustness (64). Texture-based classification hinges on two key factors: whether the features encode class differences and whether the models can exploit them (26). To challenge the model, the negative class was curated from visually ambiguous backgrounds. Of the 2,802 non-egg images, 56.8% were drawn from the Describable Textures Dataset, including cracked, flecked, and pitted surfaces that closely resemble SLF oothecae. The remaining images captured pristine and damaged steel, a relevant substrate given its prevalence on freight infrastructure. This diversity ensured the model’s exposure to realistic background textures. An exhaustive set of statistical texture features was extracted, spanning first-order (histogram-based) to higher-order descriptors such as GLCM, GLDS, Hu moments, and Zernike moments. The final feature selection identified the latter classes as dominant, offering a multiscale and complementary encoding of texture.

GLCM quantifies mid-range spatial dependencies by measuring pixel co-occurrence frequencies across orientations and distances. Statistics can be derived from the measured GLCM matrix of each image (38). Selected features such as maximal correlation coefficient, inverse difference moment, and sum of squares variance effectively captured the unique arrangement and repetition patterns of the egg mass texture. These features differentiate the granular texture of egg masses from more homogeneous or irregular backgrounds by encoding spatial correlation and local texture smoothness. GLDS features quantify local intensity variations by measuring the distribution of gray-level differences between neighboring pixels. Notably, contrast and homogeneity captured critical texture roughness and uniformity differences; egg mass textures showed higher contrast and varied homogeneity compared to non-egg regions, reflecting their characteristic granular, uneven surface. Hu moments offer invariant shape descriptors based on image moments that are robust to rotation, scaling, and translation (41). The first three Hu moments identified capture global shape properties of the texture regions, adding geometrical context to the intensity-based statistics and helping to separate the more structured egg mass shapes from background textures. Zernike moments, also rotation-invariant, decompose image patches into orthogonal polynomials to capture complex shape features (41). Selected moments at specific radii encode fine-scale geometric and symmetry information that complements the Hu moments, further enhancing discrimination between egg and non-egg textures.

Although external testing indicates that the model is not yet deployable for heterogeneous images, the current performance offers strong proof-of-concept in a controlled environment. Uniform image masking across classes, careful feature reduction, stratified validation, class weighting, and bootstrapping were employed to manage class imbalance without synthetic augmentation, which can degrade performance in high-dimensional space (65, 66). Small datasets are prone to instability (48), and SMOTE variants can introduce artifacts or collapse class variance without strong prior feature filtering. The final classifier was evaluated using confusion matrices. Both false positive and false negative rates were low, which is essential for deployment. In real-world inspection systems, false negatives risk undetected infestation, while false positives increase manual workload. The classifier’s high specificity and sensitivity suggest that texture-based models can serve as reliable and scalable screening tools in invasive species management.

One key limitation to the model remains: segmentation of egg masses from full-resolution images. Masking negative images approximates symmetry but does not eliminate the possibility of pipeline artifacts. More importantly, mask definition is still manual or semi-automated. While tools like Labkit offer some efficiency, they are not fully deployable in high-throughput settings. The external evaluation using citizen science imagery further elucidates this. The observed collapse in MCC (0.060) reflects domain mismatch rather than a failure of texture discrimination itself. The model was trained on images acquired at fixed working distance, controlled lighting, and consistent substrates, conditions under which SLF egg masses exhibit a stable and reproducible texture signature. When these acquisition constraints are relaxed, the extracted feature distributions shift, and decision boundaries learned during training no longer remain valid. Nonetheless, this work confirms that the distinctive texture signature of SLF egg masses may suffice for reliable detection, particularly on artificial substrates like trains, containers, and vehicles, where inspection is urgently needed.

## 5 Conclusion

We present a robust, interpretable framework for SLF egg mass detection using texture-based image classification. Built on a curated dataset and a comprehensive suite of spatial descriptors, the final model, a support vector machine with an RBF kernel (*C* = 5, *γ* = scale) trained on 12 features, achieves strong performance. It yielded a mean MCC of 0.881 (SD 0.037) under 5-fold stratified cross-validation, with fold-wise MCC values ranging from 0.822 to 0.939, alongside efficient training and inference. Generalization performance, evaluated on a held-out test set using bootstrap resampling (1,000 iterations), resulted in a mean MCC of 0.836 (SD 0.037; 95% CI: 0.761–0.904). While classification performance underscores the tractability of the task, the broader contribution is demonstrating that texture-based methods can deliver low-cost, interpretable, and scalable solutions for early detection.

More work is required before this technology can be generalized to external field images or deployed. The model provides image-level screening, not localized identification of SLF egg masses. Although promising, our study is further subject to two major limitations. First, all samples in this study were collected from a single population of SLF, such that variation due to geography or other biological variables is not represented in these images. The resulting correlation between such biologically similar samples may result in optimistic performance. Second, all eggs in this study were laid on roofing paper substrates, whereas substrates of greatest economic and environmental impact are vehicles and shipping containers. Egg masses laid on these substrates may physically differ. Importantly, this variation is not reflected in our controlled imaging environment. Despite these limitations, our work shows the promise of integrating textural analysis with computer vision to reduce the labor and hazards associated with manual inspection. For example, a camera mounted on a robotic chassis at international ports or inter-state borders could scan for eggs underneath a shipping or moving container, where humans cannot safely inspect, then alert a human operator to potential egg presence for follow-up inspection and egg-mass removal. Deploying lightweight computer vision systems at such transport checkpoints could alleviate reliance on labor-intensive visual surveys, reduce the risk of undetected infestations, and improve trust in screening outcomes. Future work should focus on automating segmentation in field images as well as laboratory images, embedding the model in portable inspection tools, and defining the acceptable error rates for field implementation.

## Data Availability

The datasets presented in this study can be found in online repositories. The names of the repository/repositories and accession number(s) can be found below: https://github.com/karnegre/slf-classifier/tree/master.
