# Supplementary material for: Preventing spread of the invasive spotted lanternfly via texture-based automated egg detection
Source: Front Insect Sci. 2026 Mar 23;6:1678964. doi: 10.3389/finsc.2026.1678964 (PMC13051359; doi:10.3389/finsc.2026.1678964)
Supplement: Supplementary file 1 [file Supplementaryfile1.pdf]

Supplementary Material

All data and code required to fully reproduce the pipeline are provided in the public project repository (GitHub: `https://github.com/karnegre/slf-classifier/tree/master`). For reader convenience, the exact locations of the reproducibility-relevant files are listed below.

- **Final feature set (ranks, scores, p-values):** `/outputs/feature_selection_log.txt`
- **Fixed random seeds:** lines 37–38 of `/outputs/performance_summary.txt`
- **Exact split lists and CV results:** `/outputs/gridsearch_results.csv`
- **Software versions:** `/requirements.txt`
- **Raw and processed images and masks:** `/image sets/`
- **All preprocessing, feature-extraction, and classifier scripts:** `/src/`

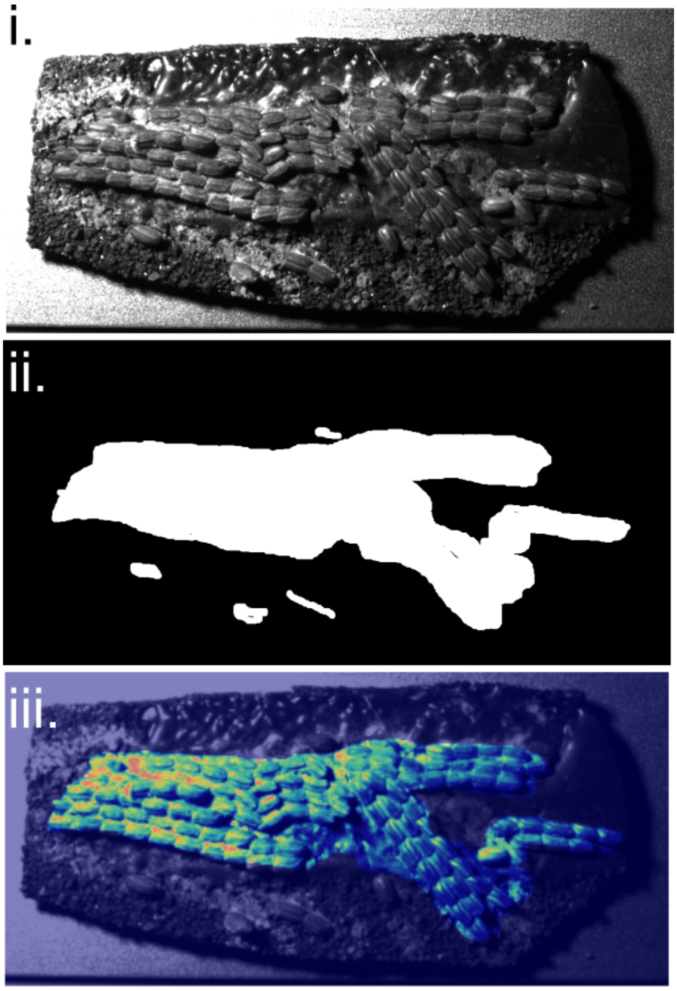

Figure S1: Segmentation workflow showing: (i) original image; (ii) segmentation using Labkit; and (iii) delineated egg mass from a complex background.

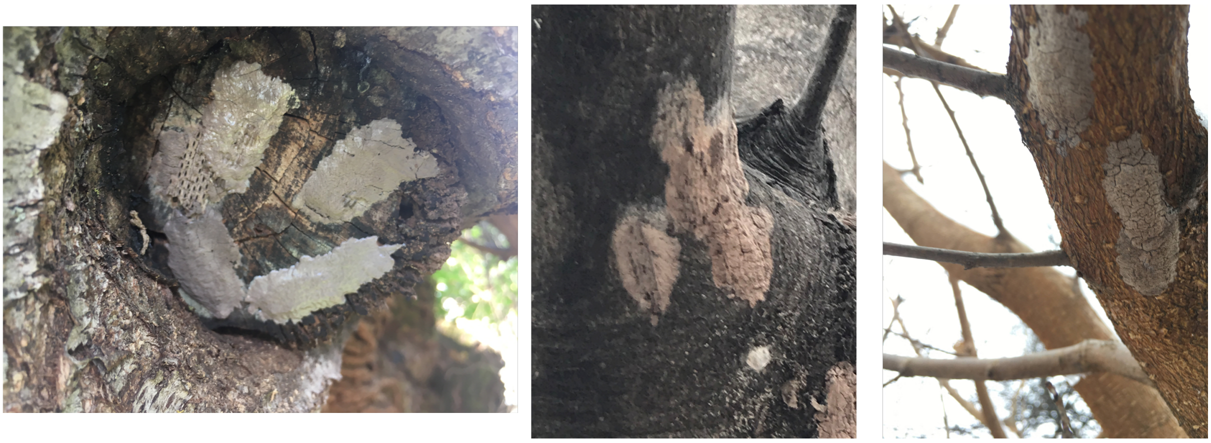

Figure S2: Representative examples from the citizen science dataset, showing three of the 251 images collected via crowdsourcing and web scraping.

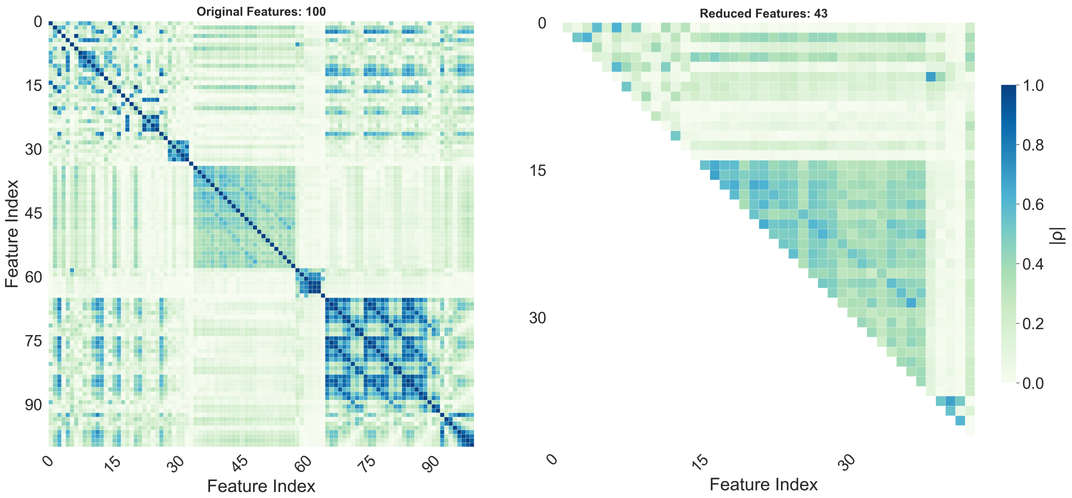

Figure S3: Hybrid filter-based feature selection. Pearson correlation filtering ($|\rho| > 0.70$) reduces 100 features to 43.

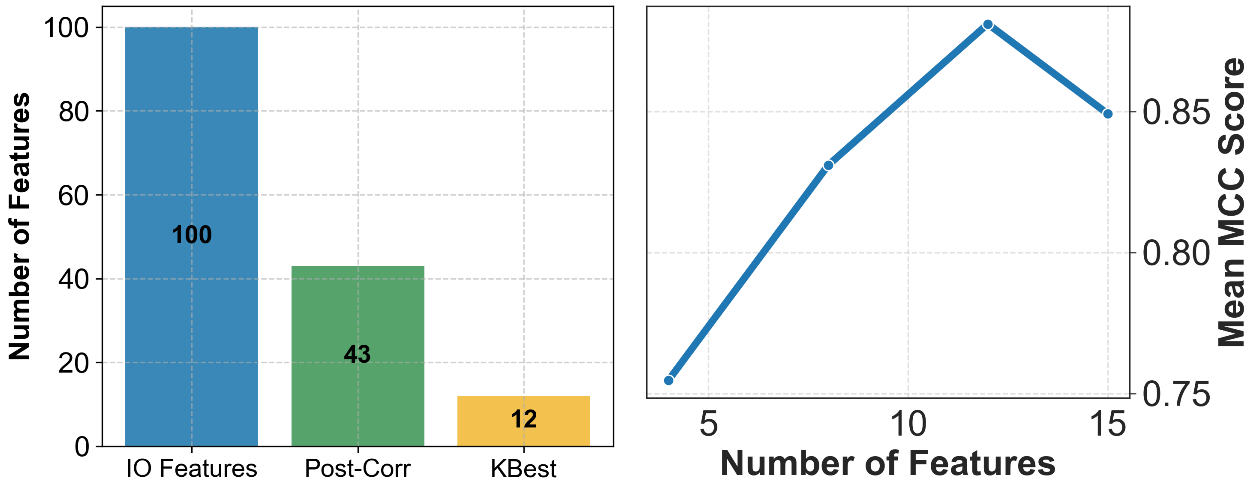

(4a) Feature reduction workflow

(4b) Model performance for k-best selection

Figure S4: Feature engineering: (a) Progressive reduction of the initial high-dimensional feature set, including removal of highly correlated features followed by further pruning via *k*-best selection during model training; (b) model performance as a function of *k*-best feature selection, showing a peak MCC at 12 features.

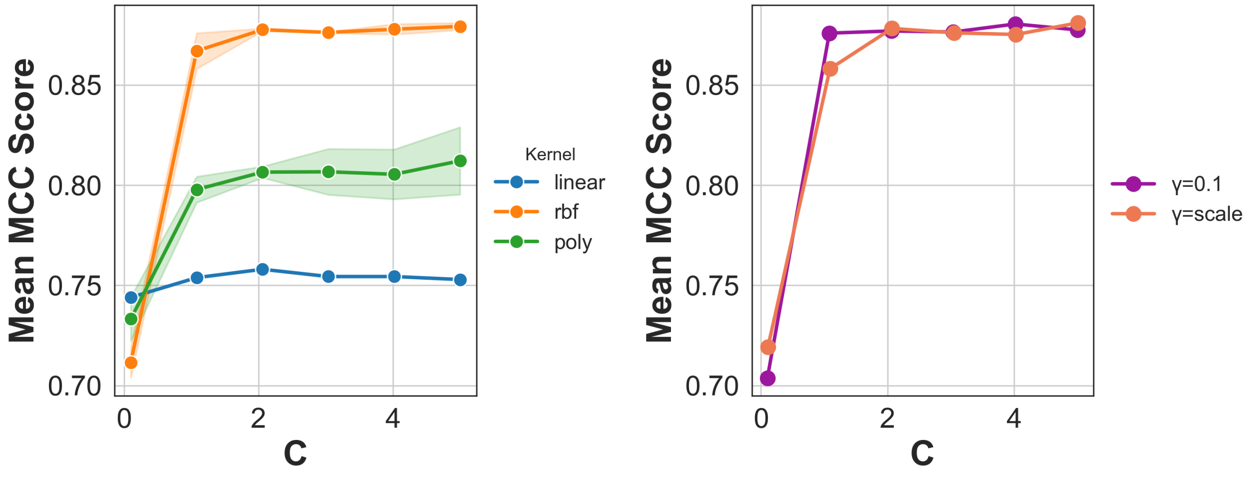

(5c) Kernel comparison across training splits

(5d) RBF kernel performance over C and γ

Figure S5: SVM hyperparameter optimization using 5-fold cCXc

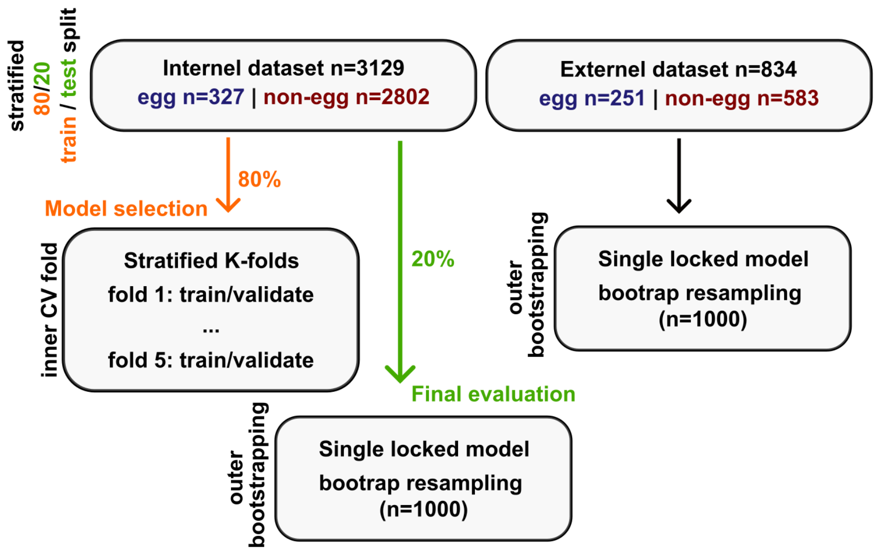

Figure S6: Overview of the data partitioning and validation strategy. The internal dataset (2,604 images: 324 egg, 2,280 non-egg) was stratified into a training set (80%) and an internal held-out test set (20%). Model selection and feature tuning were performed exclusively on the training set using 5-fold stratified cross-validation to prevent information leakage. The final locked model was evaluated on the internal test set and subsequently assessed on an independent external dataset from citizen science images not used during training or cross-validation.

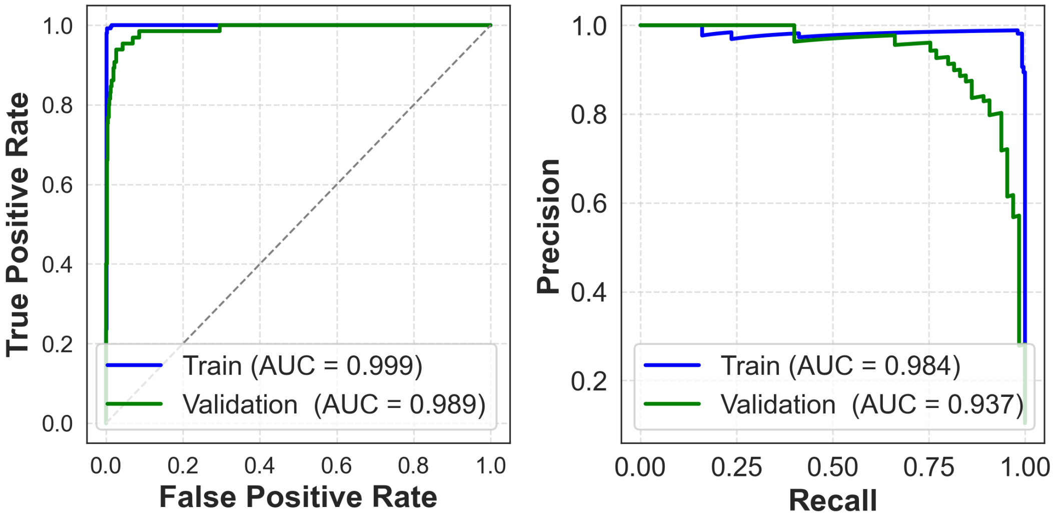

Figure S7: Model performance curves. Receiver operating characteristic (ROC) and precision-recall (PR) curves for training and validation sets confirm strong classification capacity under class imbalance.
